# Light and electron microscopic morphology of the fertilized egg and fertilized egg envelope of *Poropanchax normani*, Poeciliidae, Teleostei

**DOI:** 10.1186/s42649-022-00075-0

**Published:** 2022-07-13

**Authors:** Dong Heui Kim

**Affiliations:** Department of Convergence Medicine, Yonsei University Wonju College of Medicine, Wonju, 26426 South Korea

**Keywords:** *Poropanchax normani*, Egg envelope, Fertilized egg, Poeciliidae, Structure

## Abstract

We examined the morphology of the fertilized egg and the fine structure of fertilized egg envelopes of *Poropanchax normani* belonging to the family Poeciliidae, also known as Norman’s lampeye using light and electron microscopes. The fertilized eggs with narrow perivitelline space were found to be spherical and demersal, additionally containing small oil droplets in the vitelline membrane. Further, a bundle of adhesive filaments was observed to be present on one side of the fertilized egg. These filaments possessed remarkably high elasticity and were approximately 1-3 mm in length. The size of the fertilized egg was determined to be about 1.49 ± 0.07 mm (*n* = 30). The outer surface appeared smooth, and adhesive filaments originating at different location of the surface of the envelope were found to be distributed around the egg envelope and were joined together to form a single long bundle in scanning electron microscopic observation. A peak-like structure formed of several straight wrinkles was observed around the micropyle. However, the complete structure of the micropyle could not be studied due to the depth at which it was located. Additionally, the total thickness of the egg envelope was ascertained to be approximately12.5–14.5 μm. The egg envelope consisted of two distinct layers, an outer electron dense layer and an inner lamellar layer, further consisting of 10 sublayers of varying thicknesses. Collectively, it was observed that the morphological characteristics of the fertilized egg, fine structures surrounding the micropyle, outer surface, adhesive structure consisting adhesive filaments, and sections of fertilized egg envelope displayed species specificity.

## Introduction

Norman’s lampeye (*Poropanchax normani* Ahl, and LXXVII. 1928) is a teleost that belongs to the family Poeciliidae, order Cyprinodontiformes, and class Actinopterygii. Norman’s lampeye is named after the characteristic bright single mark located in the eye region. This species typically inhabits small rivers and swamps throughout central and western Africa (Wikipedia contributors, Poropanchax normani 2021). The maximum total length of the organism is approximately 4 cm. The males are slightly more colorful than the females. Additionally, the fins developed by males are long and pointed, while those in females are short and round (Fishkeeper contributors, Lampeye, Poropanchax normani 2021).

The fertilized egg of teleosts is surrounded by an egg envelope, which plays a crucial role in various functions such as diffusive exchange of gases, selective transport of necessary materials, protection from physical impacts and effects of chemicals, prevention of pathogenic infections, fixation to a spawning substrate in adhesive eggs, and inhibition of polyspermy through micropyle, sperm entry pore (Laale 1980; Grierson and Neville 1981; Harvey et al. 1983; Cameron and Hunter 1984). The morphology of the fertilized egg and egg envelope have been known to be influenced by various factors such as physical and chemical properties of the water environment, reproductive habits, and geographical distribution (Ivankov and Kurdyayeva 1973; Stehr and Hawkes 1979; Laale 1980; Kim et al. 1999; Kim 2020). Furthermore, the morphology of the fertilized egg, the fine structure of outer surface, micropyle, and sections of fertilized egg envelope studied have exhibited species, genus or family specificities (Kim et al. 1998, 1999; Joo and Kim 2013; Kwon et al. 2015; Choi et al. 2019; Kim 2020; Sohn and Kim 2021).

Norman’s lampeye has previously been studied in the report of new species (Ahl and LXXVII. 1928), an examination of fish fauna of northern Chad (Trape 2013), and analysis of the mitochondrial genome (Ren et al. 2021). However, there is no structural research on the egg envelope surrounding the unfertilized or fertilized egg. The lack of such research may be attributed to the difficulty of obtaining fertilized eggs from this species in laboratories, mainly owing to the small size of the organism (3–4 cm). Therefore, we have specifically focused on studying the morphology of the fertilized egg, fine structures of micropyle, the outer surface, and sections of fertilized egg envelopes to determine species specificity in Norman’s lampeye, *Poropanchax normani* belonging to Poeciliidae family, Cyprinodontiformes order, and Actinopterygii class using light and electron microscopes.

## Materials and methods

### Animals

A total of 100 Norman’s lampeye (total length: about 3-4 cm) specimens were used in this study. They were purchased from TrofishNet Aquarium (Yongin, Korea). Tap water treated with chlorine remover (AquaSafe™, Tetra Co. Ltd., Germany) was used for the rearing of the fish. The temperature and pH of the culturing water were maintained at 25 ± 0.5 °C and 7.0 ± 0.5, respectively. Biological filtration was performed using a sponge filter (Tetra TwinBrillant Super Filter™, Tetra Co. Ltd., Germany). Artificial led light (20 w) was made available for 9 h, and live *Artemia* nauplii (Sauders, U.S.A.) were provided as food three times per day. And the scraps and excrement that settled to the bottom of the aquarium were eliminated by exchanging one-fourth of the water on a daily basis to get a clean fertilize eggs without artefacts.

### Collection of fertilized eggs

For breeding, 20 adult fish were selected and moved to a glass tank (60 × 30 × 35 cm). The water used for breeding was treated with peat moss and maintained at 25 ± 0.5 °C and pH 6.0 ± 0.5. A bundle of plastic strings (each 30 cm in length) was used as a spawning substrate. The fertilized egg was isolated using fingers, while being careful not to break the fertilized eggs. The diameter of fertilized eggs confirmed to have perivitelline space (*n* = 30) were measured using a digital microscope (AD-7013MZT, Dino-Lite, Anmo, Taiwan) and used for morphological analyses.

### Electron microscopy

For observation under the transmission electron microscope (TEM), a hole was pierced in the egg envelopes of eggs that were fertilized first using a 0.33 mm needle. The purpose of this was to increase the permeability of fixers. Following this, they were fixed in 2.5% glutaraldehyde in 0.1 M phosphate buffer (pH 7.4) for 12 h at 4 °C. After prefixation, the specimens were washed twice with the same buffer solution for 20 min each. They were then postfixed in 1% osmium tetroxide in 0.1 M phosphate buffer solution (pH 7.4) for 2 h at room temperature. Specimens were dehydrated in ethanol, cleared in propylene oxide, and embedded in an Epon mixture. Ultrathin sections of embedded fertilized egg envelope were taken with an ultramicrotome (Ultracut E, Reichert-Jung, Austria) at a thickness of about 60 nm. The ultrathin sections were mounted onto copper grids, double stained with uranyl acetate followed by lead citrate, and observed using a TEM (JEM-1400, JEOL, Japan).

For scanning electron microscope observation, subsequent steps of prefixation, postfixation and dehydration were conducted by following the same procedure as that for TEM. The samples were replaced with tert-butyl alcohol and freeze dried (ES-2030, Hitachi, Japan). Specimens were coated with platinum by ion sputter (E-1045, Hitachi, Japan), and subsequently, examined using a tabletop scanning electron microscope (TM-1000, Hitachi, Japan).

## Results and discussion

### Morphology of fertilized eggs

**Fig. 1:**
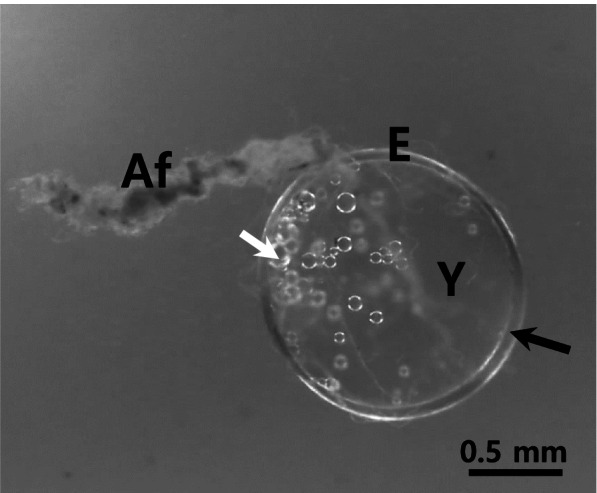
A fertilized egg of lampeye, *Poropanchax normani*. White arrow, lipid droplets; E, egg envelope; Y, yolk. The perivitelline space (black arrow) was very small and a bundle of adhesive filaments (Af) was on one side of the fertilized egg

In general, the morphology of the fertilized eggs displayed family or genus specificities, although their size and adhesive properties are typically different in fishes of families Belontiidae, Characidae, Cichlidae, Cyprinidae and Callichthyidae (Kim et al. 1996, 1999, 2005, 2009; Joo and Kim 2013; Choi et al. 2019). The fertilized egg of Norman’s lampeye was spherical and demersal, additionally consisting of a narrow perivitelline space and small oil droplets in the vitelline membrane (Fig. 1). The size of the fertilized egg was determined to be approximately 1.49 ± 0.07 mm (*n* = 30). A bundle of adhesive filaments was observed on one side of the fertilized egg, which is useful for its attachment to the spawning place. The length of the bundle of adhesive filaments having significantly high elasticity was found to be approximately 1–3 mm. The adhesive property was found to be exclusive to this bundle. It was ascertained that the adhesive property remained unaltered even after electron microscopic preparation. However, the fertilized egg itself did not possess adhesive properties. The morphology of this fertilized egg is identical to that of *Melanotaenia praecox*, which belongs to Melanotaeniidae under light microscope (Sohn and Kim 2021).

In general, the adhesive fertilized eggs may or may not possess adhesive structures in teleost. This species belongs to the family Cichlidae, the members of which have and an adhesive reticular structure on the outer surface of the fertilized egg (Deung et al. 1997; Kim et al. 2009). The tomato clown anemonefish (Pomacentridae) and dark sleeper (Eleotrididae) species, despite belonging to different families and having disparate habitats, possess long elliptical fertilized eggs that display identical morphologies as they contain a bundle of adhesive filaments (Kim et al. 1998, 2002). Peculiarly enough, the adhesive property of the fertilized eggs, lacking adhesive structures from *Ancistrus cirrhosus* is known to disappear after spawning, although it remains intact in some parts of the fertilized egg which come in contact with other eggs or the spawning substrate (Kim 2020). Additionally, *Corydoras adolfoi* and *C. sterbai* (family Callichthyidae) have similar adhesive protuberance structures on the fertilized eggs, but their ultrastuctures is different (Choi et al. 2019).

### Structure of fertilized egg envelope

**Fig. 2:**
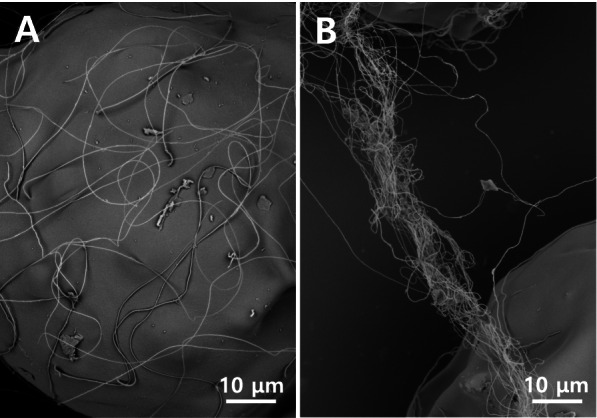
Scanning electron micrographs of the outer surface of fertilized egg envelope. The outer surface was smooth and the adhesive filaments are distributed around the egg envelope (**A**). Each adhesive filament was joined together to form a long bundle (**B**)

The outer surface of the fertilized egg envelope of *P. normani* was smooth and adhesive filaments originated at different locations were distributed around the egg envelope (Fig. 2A), each of which was joined together to form a single long bundle (Fig. 2B). In a study based on *Melanotaenia praecox* (belonging to Melanotaeniidae), the long and thick adhesive filaments were only observed at the area of the animal pole, while short and thin adhesive filaments were present around the long filament. Finally, long adhesive filaments were coiled together as observed under the scanning electron microscope (Sohn and Kim 2021).

**Fig. 3:**
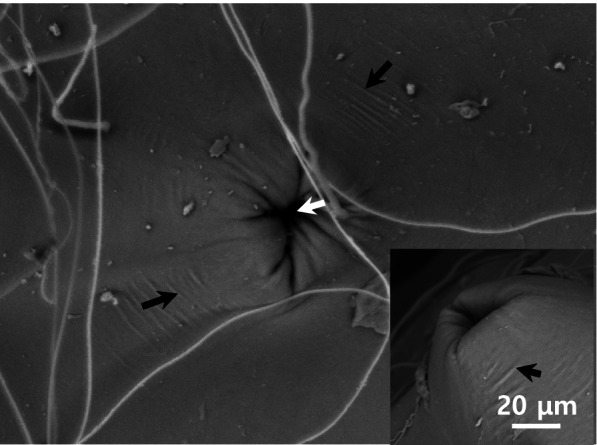
Scanning electron micrograph of micropyle (white arrow) on the fertilized egg envelopes from *Poropanchax normani*. But the complete structure of micropyle could not be observed because of the depth in which it is located. The structure around the micropyle was a peak shape with several straight wrinkles (black arrows)

**Fig. 4:**
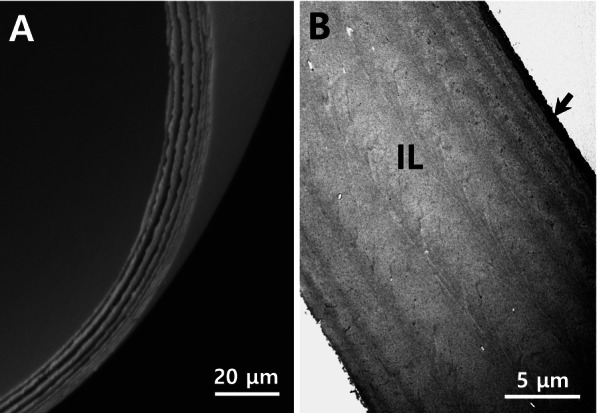
Electron micrographs of cross section of the fertilized egg envelopes. The fertilized egg envelope had a lamellar layered structure under the scanning electron microscope (**A**) and the fertilized egg envelope consists of two layers, an outer electron dense layer (arrow) and an inner lamellar layer (IL) comprising of 10 sublayers of varying thicknesses under the transmission electron microscope (**B**)

A distinct, peak-like structure consisting of several straight wrinkles was observed around the micropyle (Fig. 3). However, the complete structure of the micropyle could not be observed due to the depth at which it was located. The micropyle of *M. praecox* was known to be conical in shape, having whippy, adhesive filaments. This occurrence holds true although the fertilized eggs of *M. praecox* and Norman’s lampeye have the same external morphology under the light microscope (Sohn and Kim 2021). Additionally, the total thickness of the egg envelope was ascertained to be approximately12.5–14.5 μm. The fertilized egg envelope consisted of two distinct layers, an outer electron dense layer and an inner lamellar layer, further consisting of 10 sublayers of varying thicknesses (Fig. 4A & B). The morphology of the fertilized egg and sections of fertilized egg envelope are remarkably similar with that of *M. praecox*, which belongs to a different family. However, the inner sublayer of *M. praecox* consisted of 8 layers (Sohn and Kim 2021).

The adhesive or non-adhesive structures found on the outer surface of the fertilized egg envelope displayed species, genus or family specificities. A majority of species belonging to Cyprinidae possess different structures: The structures present in the fertilized egg envelopes in *Tanichthys alborubes* have a rod-like appearance (Kim et al. 1998), while *Zacco platypus* has Indian club-like structures (Deung et al. 2000). Further, *Hemibarbus longirostris* possesses taste bud-like structures (Kim et al. 2001) and *Danio rerio* shows structures that appear knob-like (Joo and Kim 2013). Adhesive, reticular structures are present in Cichlidae (Deung et al. 1997), while in Belontiidae, numerous grooves of the envelopes covered by thin adhesive layers may be observed (Kim et al. 1999). The family Nothobranchiidae characteristically has several adhesive whip-like structures (Kwon et al. 2017). Finally, the Callichthyidae exhibits adhesive protuberances (Choi et al. 2019). Therefore, it may be concluded that the aforementioned families have shown family specificity. Also, it may be noted that these outer surface structures may be either identical or different, regardless of their belonging to different genera (Kim et al. 1996, 2005). However, it was ascertained that despite tomato clown anemonefish and dark sleeper belonging to different families, i.e., Pomacentridae (Kim et al. 1998) and Eleotrididae (Kim et al. 2002) respectively, they have displayed an identical smooth outer surface of the fertilized egg envelopes, such as Norman’s lampeye.

Species, genus, or family specificities such as the structures on the outer egg envelope seem to play a salient role in the structure of the micropyle and the passage of sperm without acrosome. The various species belonging to the Characidae and Belontiidae, along with those of the genus *Nothobranchius*, show an identical micropyle structure within the same family (Kim et al. 1996, 1999; Kwon et al. 2017; Chang et al. 2019). However, the morphology of micropyle differs according to the species in Cyprinidae (Kim et al. 1998, 2001; Deung et al. 2000). The micropyle is not found to be present on the fertilized egg with a bundle of adhesive filaments (Kim et al. 1998, 2002) but that of Norman’s lampeye was found to be outside of the bundle of filaments.

In general, the fertilized egg envelope of fish eggs was found to consist of two or three layers. In particular, the inner layer was seen to have a layered structure consisting of several sublayers. The number of sublayers present in the inner layer was found to vary even within the same family (Kim et al. 1996, 2005; Chang et al. 2019; Sohn and Kim 2021). However, the section of the fertilized egg envelope has the structure specific to all species belonging to Belontiidae (Kim et al. 1999), Cichlidae (Deung et al. 1997; Kim et al. 2009), Callichthyidae (Choi et al. 2019), and Nothobranchiidae (Kwon et al. 2015, 2017). The thickness of the fertilized egg and the number of sublayers present in the inner layer cannot be measured using different approaches without an accurate cross section. Also, the total number of fertilized egg envelopes can be measured differently using a transmission electron microscope and scanning electron microscope. The current study is limited in that it focuses on a single species. Hence further research involving the various species belonging to Poeciliidae is required to gain more insight into the structure of the fertilized egg and the fertilized egg envelope.

## Conclusions

The fertilized eggs of Norman’s lampeye, *Poropanchax normani* belonging to Poeciliidae were spherical and demersal, additionally consisting of a narrow perivitelline space and small oil droplets in the vitelline membrane. Furthermore, a bundle of adhesive filaments possessing remarkably high elasticity was present on one side of the fertilized egg. Their primary function seemed to be attachment on the spawning place or other structures. The adhesive filaments originating at different location on the surface of the envelope are distributed around the egg envelope, each of them being joined together to form a single long bundle. The outer surface was found to be smooth. A peak-like structure formed of several straight wrinkles was observed around the micropyle. Also, the fertilized egg envelope consisted of two distinct layers, an outer electron dense layer and an inner lamellar layer, further consisting of 10 sublayers of varying thicknesses. These morphological characteristics of the fertilized egg and fine structures surrounding the micropyle, outer surface, adhesive structure consisting of adhesive filaments, and sections of fertilized egg envelope have demonstrated species specificity of *Poropanchax normani.*

## Data Availability

No applicable.

## Abbreviations

TEM: Transmission electron microscope
